# Small Animal Video Tracking for Activity and Path Analysis Using a Novel Open-Source Multi-Platform Application (AnimApp)

**DOI:** 10.1038/s41598-019-48841-7

**Published:** 2019-08-26

**Authors:** Srinivasa R. Rao, Sam W. Z. Olechnowicz, Patrick Krätschmer, James E. C. Jepson, Claire M. Edwards, James R. Edwards

**Affiliations:** 1 Botnar Research Centre, Nuffield Department of Orthopaedics, Rheumatology and Musculoskeletal Sciences, University of Oxford, Oxford, UK; 2 Nuffield Department of Surgical Sciences, University of Oxford, Oxford, UK; 3 Department of Clinical and Experimental Epilepsy, UCL Institute of Neurology, London, UK

**Keywords:** Software, Animal behaviour

## Abstract

Experimental biological model system outcomes such as altered animal movement capability or behaviour are difficult to quantify manually. Existing automatic movement tracking devices can be expensive and imposing upon the typical environment of the animal model. We have developed a novel multiplatform, free-to-use open-source application based on OpenCV, called AnimApp. Our results show that AnimApp can reliably and reproducibly track movement of small animals such as rodents or insects, and quantify parameters of action including distance and speed in order to detect activity changes arising from handling, environment enrichment, or temperature alteration. This system offers an accurate and reproducible experimental approach with potential for simple, fast and flexible analysis of movement and behaviour in a wide range of model systems.

## Introduction

The majority of experimental *in vivo* surgical models or animals generated following genetic manipulation lead to physiological effects impacting behaviour and locomotion^1^. Induction of chronic pain or light sensitivity, for example, may represent a useful outcome in an experimental model of disease, but these behaviours are difficult to quantify without the aid of automated animal tracking devices and complex analytical systems. Such investigations are reliant on, and often limited by, the selection of an appropriate method of assessment.

Existing methods compromise between the quality of motion data collected, ease of setup of the chosen device and the overall level of throughput. Infrared movement sensors can be deployed for long periods of time with minimal supervision, but only provide low detail information about average rodent activity per home cage^2^. In contrast, use of multiple high-resolution CCD cameras provides high quality recording and analysis of movement such as the detection of subtle gait abnormalities, but this approach does not lend itself to high throughput analysis of mice^3^. Similar strategies exist to quantify insect locomotion at either the larval or adult stage, ranging from manual counting of grids crossed^4^ to automated counting of infrared beam crosses^5^ and automated video tracking^6–8^. Use of a dedicated filming stage for rodents or insects allows for clear detection but potentially disturbs normal behaviour while further increasing barriers-to-entry^9^, but may be necessary for detection of behaviours such as grooming^10^. Alternatively, filming natural movement in the home cage currently requires a dedicated computer and cage setup^11^. Established institutions which focus on small animal behaviour may invest in such complex systems, however there are few methods available for researchers without dedicated infrastructure to easily and flexibly test for reproducible changes in movement or behaviour.

Here we describe movement capture of small animals in their home environment, and analysis using a novel open-source OpenCV-based application called AnimApp. This system uses low-cost equipment which is easy to set up and mostly already available to the average animal researcher. The mobile version of the app, targeted at Android smartphones, is very simple to install and use, and does not require processing of files on a standalone computer, while a desktop (PC/Mac/Linux) version is also available for batch processing of videos from any digital recording device. We find that mice are highly active when using the home cage as a stage, meaning that this approach is fast (2 minutes per mouse) and results in reproducible detection of altered activity. AnimApp tracking of insect larvae movement and contractions further highlights the adaptability of this system for use with a wide range of animal model organisms and output criteria.

## Results and Discussion

**Figure 1:**
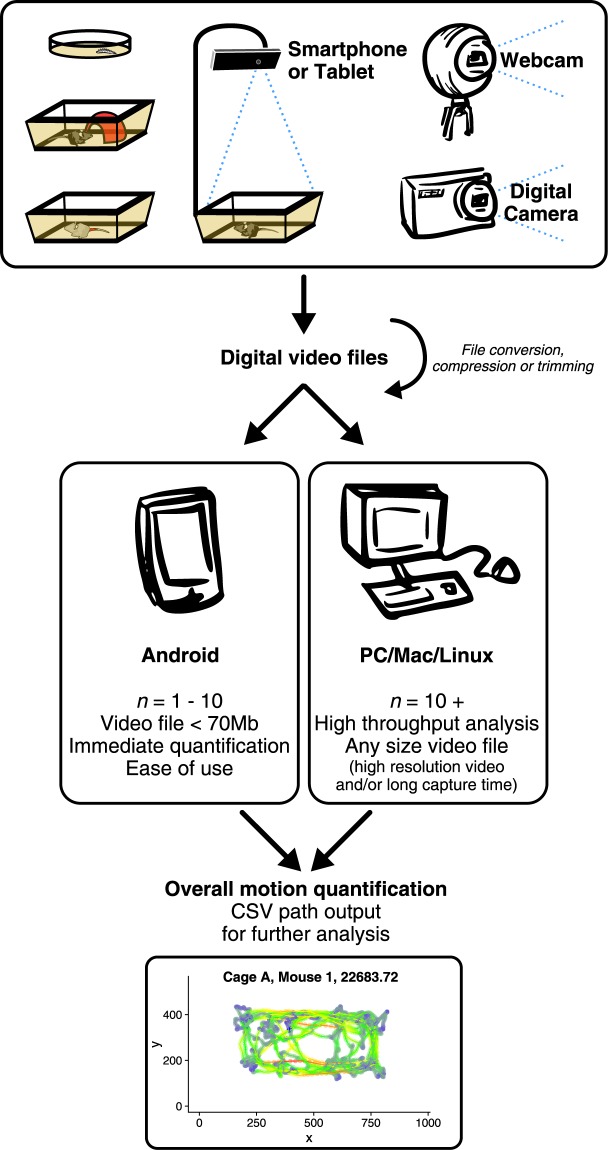
Graphical abstract. Digital video captured from a variety of possible sources can be analysed by the AnimApp program directly on an Android-compatible smartphone or tablet for small-batch files, or transferred to a computer for video trimming and conversion, and high-throughput high-resolution analysis. AnimApp running on either platform gives total movement summary and .csv formatted tracked path for further analysis in other software (example of tracked path processed with colouring representing instantaneous velocity, using R and ggplot2^19,20^).

This research project demonstrates how an animal or object with sufficient background contrast can be digitally filmed and assessed using AnimApp, as depicted in the workflow example (Fig. 1). Three distinct experimental model systems common in laboratory and pre-clinical research (*Drosophila melanogaster* adult flies and larvae, white mice with added colour contrast, and black mice alone or in the presence of stimuli such as housing), were employed to develop and validate the application for use in assessing parameters of behaviour and activity *in situ*.

**Figure 2:**
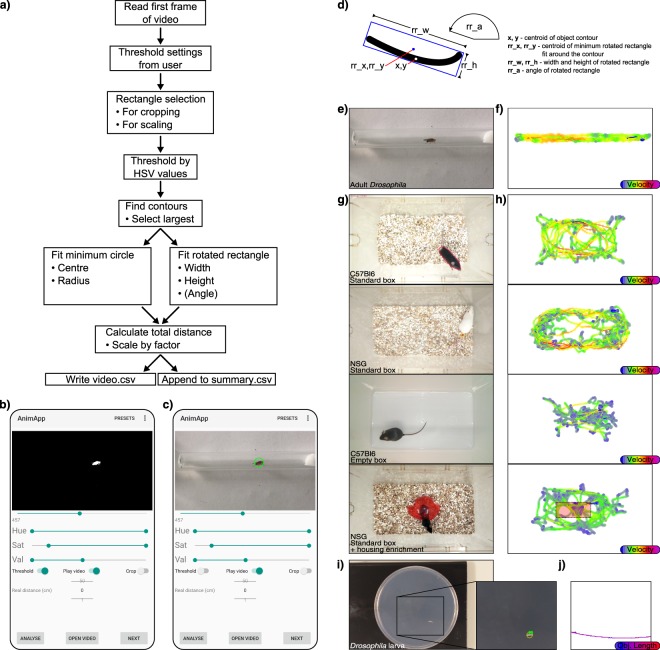
(**a**) Flowchart for AnimApp function for analysis of recordings. Upon opening a video file in AnimApp, the first frame is shown as a preview. A green marker square can be drawn for video cropping (‘Crop’ switch), or alternatively for reference to a known scale such as a visible ruler. (**b**) Activating the “Threshold” switch allows precise setting of the Hue, Saturation and Value (HSV) sliders in order to set the colour threshold for animal detection. The slider under the preview frame can be moved to test the threshold settings frame-by-frame throughout the video. Preset HSV thresholds can be loaded from the “Presets” tab (see Supplemental Fig. 1,a). (**c**) Resulting individual animal detection indicated by green circle. Tapping ‘Play Video’ runs the video during analysis to check detection accuracy, while tapping ‘Next’ proceeds to analyse the video. (**d**) Example object contour and labels describing AnimApp.csv output columns. (**e**) Example adult Drosophila video and (**f)** path tracking, with colour indicating relative instantaneous speed. (**g**) Mouse detection in home cage or empty transport box, with white mouse detected by a red pen marking at the base of the tail, and a representative translucent red housing enrichment object. (**h**) Resulting path traces of the respective animals in (**f**) with the processed path colour indicating instantaneous speed. The translucent red square indicates the “centre of the box” area used in calculations for Fig. 3f. (**i)** Equivalent setup for tracking a white Drosophila larva over a dark agar plate. (**j**) Plot of larvae path, with path colour indicating object (larval) length at each frame.

Recording with a smartphone was found to offer greater flexibility in recording parameters along with the availability of direct processing within the device through the AnimApp application. Alternatively, video files recorded by webcams or other cameras could be transferred and processed using the desktop version of AnimApp. Longer videos and larger datasets were analysed using the desktop version in order to take advantage of increased processor speed and batch processing. AnimApp quantifies total movement overall, as well as creating an individual record detailing the path taken, as well as animal size and shape at each frame. An overview of AnimApp function is depicted in Fig. 2a. Using the first frame of a video file, AnimApp allows the user to crop the image and set a scale of known size by dragging a rectangle over the image. Optimising and setting of colour threshold based on Hue, Saturation and Value (HSV) colour space (Fig. 2b) allows AnimApp to find the desired object within the field of view for path tracking (Fig. 2c). Common thresholding settings are pre-installed, while user-defined thresholding values can be loaded from the “Presets” menu (Supplementary Fig. 1a), and exported for sharing to aid in reproducibility. As AnimApp requires a HSV colour difference in order to detect an object over a background, careful experimental setup with respect to lighting, filming distance and video capture techniques are necessary. With ease of use in mind, we have developed our software to accept several video types as input. In dark environments, or for fast-moving small animals, other video sources such as infrared, night-vision, or high-speed digital cameras may be used, and the video files imported to computer or mobile device for analysis in AnimApp. However, all animal tracking presented in this paper was successfully performed with standard lab lighting and in-built mobile phone camera.

After frame-by-frame analysis, AnimApp generates a plot of the tracked object, with a slider allowing inspection of the path frame-by-frame through the video, and a measurement of total distance travelled to the frame selected. A comma-separated variable file (.csv file) named *AnimApp_summary*.*csv* provides a log of each analysis with the number of video frames, distance moved and scaling factor applied. AnimApp also generates a threshold settings log file (labelled *Video*.*mp4*.*settings*.*txt*) and an individual path file (*Video*.*mp4*.*csv*) containing information regarding the object at each frame (object centroid, frame number, and centroid, width and length of minimum rotated rectangle, described in Fig. 2d). This information can be further processed in other analysis software. A video of an adult *Drosophila* is provided as an example of simple animal detection and tracking in a standard clear glass tube as in the *Drosophila* ARousal Tracking system^8^ (Fig. 2e), with instantaneous object velocity calculated for path colour (Fig. 2f). Locomotion distance binning is available in app, and can be adjusted by selecting the bin size within the “Presets” tab (Supplementary Fig. 1a), and example output for this video are shown for bin width 10 and 25 (Supplementary Fig. 1b,c). This example *Drosophila* video file, a threshold preset file and an R script for calculation and display of path with velocity are supplied as Supplementary Files.

For rodent filming, home cages were opened and enrichments removed, before recording mice individually from a suspended smartphone (Fig. 2g). Prior to filming, mice were handled indirectly using a cardboard tube in order to reduce animal anxiety, as described previously^12^. For initial optimisation, black C57Bl6 and white NOD/SCID-GAMMA (NSG) mice were used. The strong contrast of C57Bl6 mice to the white bedding background allowed accurate detection directly, while the NSG mice required colour marking on the base of their tail with permanent marker to increase contrast before recording. Mice were primarily filmed in isolation in their own home cages, after removal of loose bedding and enrichment objects, leaving only chip flooring. When using the home cage as a recording stage, mice frequently displayed digging and searching behaviour, as well as rearing to the cage walls, but spent minimal time grooming or otherwise stationary. Mouse behaviour was notably altered when a clean empty transport box was used as a recording stage (tracked paths shown respectively in Fig. 2h). *Drosophila* larvae were placed individually on clear petri dishes containing 2% agar, over a black background (Fig. 2i,j). We have also tested and successfully tracked other insects such as ladybirds (Supplementary Fig. 1d,e), as well as animal features (Supplementary Fig. 2), indicating that AnimApp can be used to track a wide range of targets.

**Figure 3:**
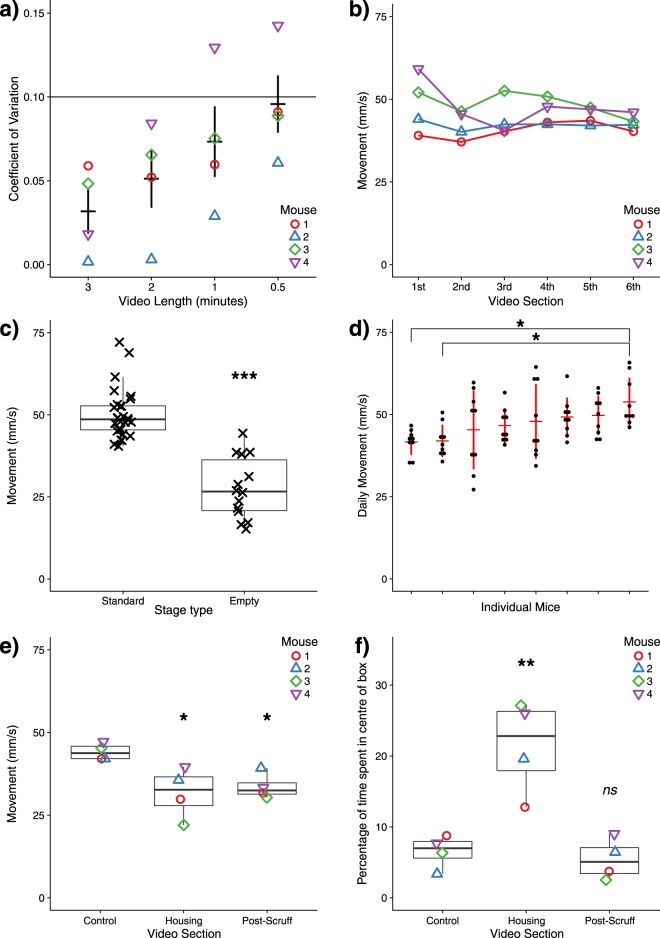
(**a**) Coefficient of variation for C57Bl6 mouse movement arising from videos of indicated length, relative to a master 6-minute video. (**b**) Individual mouse movement per minute segment of master video. (**c**) Movement of C57Bl6 mice analysed in home cage (‘standard’) or a clean ‘empty’ transport box (see Fig. 2g for examples), using 2-minute videos. (**d**) Daily variation in movement of individual control NOD/SCID-GAMMA mice, filmed biweekly for 2 minutes each session, organised by increasing mouse activity. (**e**) Overall C57Bl6 mouse movement and (**f**) percentage of time spent in centre of cage using initially a control standard filming stage, followed by introduction of a translucent red house, and following manual handling and scruff. Statistics: (**c**) Welch’s two-sample unpaired t-test; (**d–f**) One-way ANOVA with Tukey multiple comparisons, ns: not significant; *p < 0.05; **p < 0.01; ***p < 0.001.

The length of each filming session for C57Bl6 mice was optimised to balance accuracy of measurement with time efficiency. Since the animals do not move evenly throughout a recording session, short videos are prone to high measurement variation. By taking a single 6 minute source video of each mouse, and splitting this into 3, 2, 1 and 0.5 minute segments, we analysed the coefficient of variation arising from different video durations. 2 minute sections produced less than 10% variation in movement quantification compared to the complete 6 minute source (Fig. 3a), which we considered acceptable, however less active animals may require longer video durations to achieve a similar level of accuracy.

To determine whether the activity of the subject mice changes as the animals become accustomed to the presence of the camera, movement quantification from the 1 minute segments of the source videos were assessed in chronological order (Fig. 3b), and indicated no significant change in activity level throughout filming. However, more consistent results are seen in the later minutes of recording, suggesting that shorter duration videos may be equally accurate if a period of acclimatisation to the recording stage is extended.

As the filming environment would be expected to alter rodent behaviour, 2 minute videos taken of C57Bl6 mice using a “standard” home cage were compared to those filmed in completely clean “empty” transport boxes of similar dimensions. Mouse behaviour was considerably altered in the empty transport box, evidenced by significant reduction in movement distance (Fig. 3c). This indicated that for quantifying maximum movement ability, the use of the animals’ own home cage as a recording stage is superior to an empty stage. However, experimental designs which focus on interventions to increase exploratory behaviour or confidence may find benefit in using a clean sterile recording stage. These findings indicate that our analysis system is capable of detecting subtle changes in behaviour arising from an altered environment.

Movement of individual NOD/SCID-GAMMA mice was analysed through repeat recordings over the course of 6 weeks to test reproducibility of movement for individual animals between filming days (Fig. 3d). Surprisingly, reproducible differences between cage-mates are detectable, suggesting subtle differences between each individual in baseline disposition to movement.

To test the flexibility of this method, our AnimApp system was used to measure behavioural changes in response to the introduction of an enrichment object to the home cage stage and direct animal handling (“scruffing”). Tracking of rodent interaction with novel objects has been described in open field tests^9^, while direct animal handling has been reported to have an effect on anxiety-related behaviour^12^. Mouse activity was measured for 2 minutes before and immediately after introduction of red transparent housing to the centre of the home cage, then removal of the housing was followed by the mouse being scruffed for 10 seconds, during which time the animal was held inverted to simulate an experimental procedure such as intraperitoneal injection. Filming then resumed for a further 2 minutes (Fig. 3e). Notably, the introduction of housing and the handling-induced anxiety both caused a reduction in total mouse movement. To further characterise mouse behaviour in each condition, the total percentage of time spent in the centre of the recording stage was also determined by detailed analysis of AnimApp path output (Fig. 3f), and revealed a significant attraction of the animals to the red housing unit. Further analyses of behaviour such as interactions with specific locations, local environments within the cage, or even interactions between two different animals are possible with careful analysis of output path files. This highlights how AnimApp can be used for quantifying behavioural changes in terms of overall animal activity, path taken and interactions made by each individual animal. As with any behavioural study, this quantification does not discriminate between the causes of such changes, so careful experimental design is required to interpret the results.

**Figure 4:**
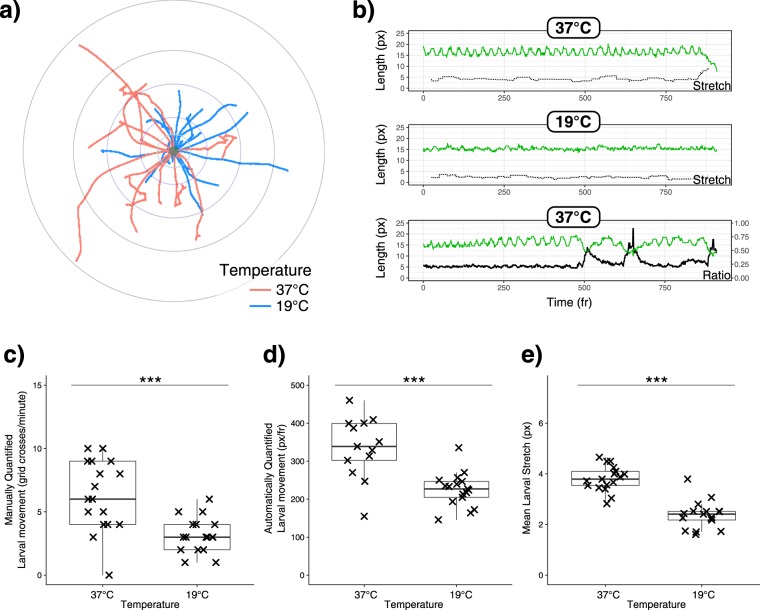
(**a**) Normalised paths taken by *Drosophila* larvae incubated at 37 °C (red) or 19 °C (blue). (**b**) Example traces of larval length (green), mean larval stretch amplitude (also known as “striding”, dashed line) and the ratio of larval length:width (heavy black line, on secondary y-axis) over time (frames) from representative videos of larvae incubated at 37 °C or 19 °C. (**c**) Manual quantification of distance travelled in 1 minute, by counting of grid-line passing events, and (**d**) using AnimApp for automatic quantification of distance in the same videos. (**e**) Mean striding or contractile length for each condition using AnimApp output. Individual larval measurements are plotted over a Tukey boxplot. Statistics: Welch’s two-sample unpaired t-test, *p < 0.05; **p < 0.01; ***p < 0.001.

The AnimApp application was also investigated for use in the assessment of movement of individual *Drosophila* larvae in a petri dish, using 1-minute videos of larvae inside an incubator set at 19 °C or 37 °C. Overlaying output movement paths normalised to the starting location provided a method of readily visualising differences (Fig. 4a). Larval contractions, also known as strides, have previously been used as a measurement of locomotion behaviour^6^. By using the length and width of the larva throughout the recordings, larval contractions could be assessed (denoted by green line, Fig. 4b), followed by further processing to determine the mean amplitude of larval contractions (dashed line, Fig. 4b). Turning larvae can be detected by increases in the ratio of larval length to width (black line, Fig. 4b). The standard method of manually quantifying larval movement, by counting grid line crossing events (Fig. 4c) was compared to automatic locomotion quantification by AnimApp (Fig. 4d). Both methods provided similar results, showing significantly lower average movement in 1 minute for larvae incubated at 19 °C compared to 37 °C (*p* < 0.001 for both methods). AnimApp was much faster to use than manual grid counting, and more precise in detecting movement, resulting in a reduced variance of data points. Mean contraction amplitude was also significantly suppressed by 19 °C incubation (*p* < 0.001, Fig. 4e).

To explore tracking of multiple colour tags within a single video, we used AnimApp to analyse a video published previously for M-Track, an app designed to detect grooming behaviour in mice^10^. The video shows a white mouse with paws labelled with red (right paw) or blue (left paw) colouring. By running the video on AnimApp with appropriate thresholding for different colours, individual detection of each paw and the whole mouse body were successful. In comparison to the published results by M-Track, AnimApp produces accurate results showing very similar patterns of motion for each paw (Supplemental Fig. 2).

## Conclusion

The AnimApp software provides a novel technique for the low-cost analysis of movement and behavioural responses in small animal research, using model organisms ranging from rodents to *Drosophila*. AnimApp outputs the total distance movement and time of the input video, frame-by-frame location of the subject, along with width, length and angle information with scaling to convert pixel distances to millimetres. Further analysis of path is flexibly performed using software such as R, example scripts for which are available in the Supplementary Methods. Using a home cage filming stage, AnimApp was able to detect subtle changes in mouse activity arising from the introduction of an enrichment object, manual handling, and even baseline variation between individual animals. The use of a home cage as a recording stage was also shown to increase activity in rodents, allowing for sensitive detection of any reductions in maximal animal activity. The shape of the detected animal can be further analysed to give information about strides and turning, particularly when using a model organism with a consistent dorsal profile, such as *Drosophila* larvae, while creative camera and recording stage setup could allow for quantification of movements in other axis directions, such as burrowing or rearing. AnimApp allows for the combination of recording and activity processing in a single smartphone or tablet, providing a fast and easy measure of activity, while further post-processing can be performed with the detailed path output files generated in AnimApp.

Tracking of multiple animals or animal features within the same video is a difficult problem, particularly in animals such as mice that frequently cross paths with one another. Other software have devised methods to track multiple animals^13–15^. The motivation of the current work was to provide a mobile device-based user-friendly interface without a steep learning curve or complex software dependencies, while being able to track individual movement accurately. However, we show that animal parts tagged with distinctly different colours can be accurately tracked, giving comparable results to dedicated software^10^. Such a method could in principle be extended to track multiple animals in AnimApp by marking each with unique colours, and processing for each colour individually in AnimApp. Merging this resulting data could provide an accurate analysis of multiple animal paths in the same video.

Potential indications for the AnimApp assessment approach include disease or genetically modified models, quantification of movement ability as a surrogate for pain, attraction or repulsion to food or other chemicals, light sensitivity or general anxiety. The raw path output also allows for quantification of attraction or repulsion from sources, and for further detailed mapping of movement path, for example if used with a maze filming stage. However, animal activity can be altered positively or negatively by both noxious or enriching stimuli, so interpretation of any results must take into account experimental design and comparison to other established complementary methods.

This study describes the development and validation of a new, free-to-use open source program aimed at reducing researcher time, improving experimental design and refining current techniques common in preclinical model studies assessing behaviour and activity outcomes. The incorporation of the AnimApp program into commonly used hand-held devices offers a reliable, cost effective and minimally invasive approach to the study of a variety of experimental model systems, in particular those where alterations in movement are indicative of specific causation, e.g. pain, neurological disease, muscle weakness.

## Equipment and Methods

### Animals

All animal experiments were conducted in accordance with the Animals Scientific Procedures Act of 1986 (UK) under UK Home Office Project License 30/2996 and PCCCC8952. Protocols were approved by the Animal Welfare and Ethical Review Body of the University of Oxford.

C57Black/6 (C57Bl6) and NOD/SCID-GAMMA (NSG) mice were routinely housed in individually ventilated cages in adherence to Home Office guidelines. Mice were transferred between recording stages and transport boxes without direct handling, by use of a cardboard tube. All *Drosophila* used were of isogenic background (iso31). To collect L3 larvae, 20% sucrose solution was poured into vials containing iso31 larvae, and after 15 minutes of incubation, floating L3 larvae were collected, washed with distilled water, gently dried, and placed onto petri dishes containing 2% agar. These petri dishes were then placed into incubators at the desired temperature.

### Video recording

Videos were filmed using an Android mobile phone, at 1920 × 1080 resolution, 29 frames/second (rodents) or at 640 × 480 resolution, 15 frames/second (larvae), using the open-source Open Camera Android app. Where necessary, video resolution was reduced, or audio removed from recorded video using ffmpeg v2.8.14-0ubuntu0.16.04.1. The camera or mobile phone was mounted on a flexible “Gooseneck”-style mounting arm with spring clamp, for easy positioning. In cases where poor contrast was expected between the animal and the background (e.g., a white mouse), coloured Sharpie permanent markers were used to make a 0.5 cm long mark at the base of the tail. A 30 cm ruler was placed in the video to give a scale measure for conversion of pixels to centimetres.

### Video analysis

Video files in a common format such as MP4 or AVI were read frame-by-frame using a Python (desktop) or Java (Android) framework (leveraging the OpenCV^16^ or JavaCV^17^ libraries respectively). Each frame was then thresholded by HSV values provided by the user at the command-line (desktop) or using a graphical user interface (Android). From the thresholded frame, contours were identified (OpenCV uses the method developed by Suzuki *et al*.^18^ internally) and the position and dimensions of the largest contour were recorded in a comma separated variable (CSV) file for plotting and further analysis. Euclidean distance between consecutive points was calculated as follows and summed to give the total distance travelled.

1 $$\sqrt{{({x}_{1}-{x}_{2})}^{2}+{({y}_{1}-{y}_{2})}^{2}}$$

(*x*_1_, *y*_1_) – *initial location of the object*.

(*x*_2_, *y*_2_) – *location of the object in the consecutive video frame*.

All source code used for this video analysis are freely available for download. The code can be downloaded from https://github.com/sraorao/animapp_desktop for the desktop version (also available as a pre-packaged Docker container for the Ubuntu platform), and from https://github.com/sraorao/AnimApp for the Android version. Instructions for installation are provided in Supplementary Data and at the respective web pages. AnimApp Android is also available for free installation via the Google Play store at https://play.google.com/store/apps/details?id=com.oxford.srao.animapp. Android app output (.csv) and settings (.txt) files are generated within the internal storage “Download” folder, for ease of location.

Post-processing of movement paths was performed using R and visualised with the ggplot2 package^19,20^, with colouring relating to animal velocity or larva length (see Supplementary Data Files). Instantaneous velocity was calculated as an average of distance travelled per 10 consecutive frames, equivalent to 333 ms. R scripts for calculating and plotting these variables are provided as Supplementary Files. For manual quantification of larval movement, the same videos that were analysed automatically were loaded on a desktop computer and a semi-transparent grid was overlayed on top. Events of larvae passing through a grid line were counted, in a method reflecting the current standard assay for locomotion in larvae^4^. For larval contraction analysis, the largest dimension of the identified contour was considered the length of the larva, and the ratio of the largest and smallest dimensions was used to distinguish movement-related contractions (strides) or changes in direction (turns).

### Statistics

All statistical tests were performed in R 3.4.2^19^. Comparison across two groups was performed using Unpaired Student’s *t-*test with Welch’s correction, and using ANOVA followed by Tukey’s test for multiple comparisons.

## Supplementary information

Supplementary info

Supplementary Info
