# Supplementary material for: Small Animal Video Tracking for Activity and Path Analysis Using a Novel Open-Source Multi-Platform Application (AnimApp)
Source: Sci Rep. 2019 Aug 26;9:12343. doi: 10.1038/s41598-019-48841-7 (PMC6710427; doi:10.1038/s41598-019-48841-7)
Supplement: Supplementary file 1 — Supplementary info [file 41598_2019_48841_MOESM1_ESM.docx]

**Small Animal Video Tracking for Activity and Path Analysis Using a Novel Open-Source Multi-Platform Application (AnimApp): Supplementary Data**

Srinivasa R Rao, Sam W Z Olechnowicz, Patrick Krätschmer, James E C Jepson, Claire M Edwards, James R Edwards


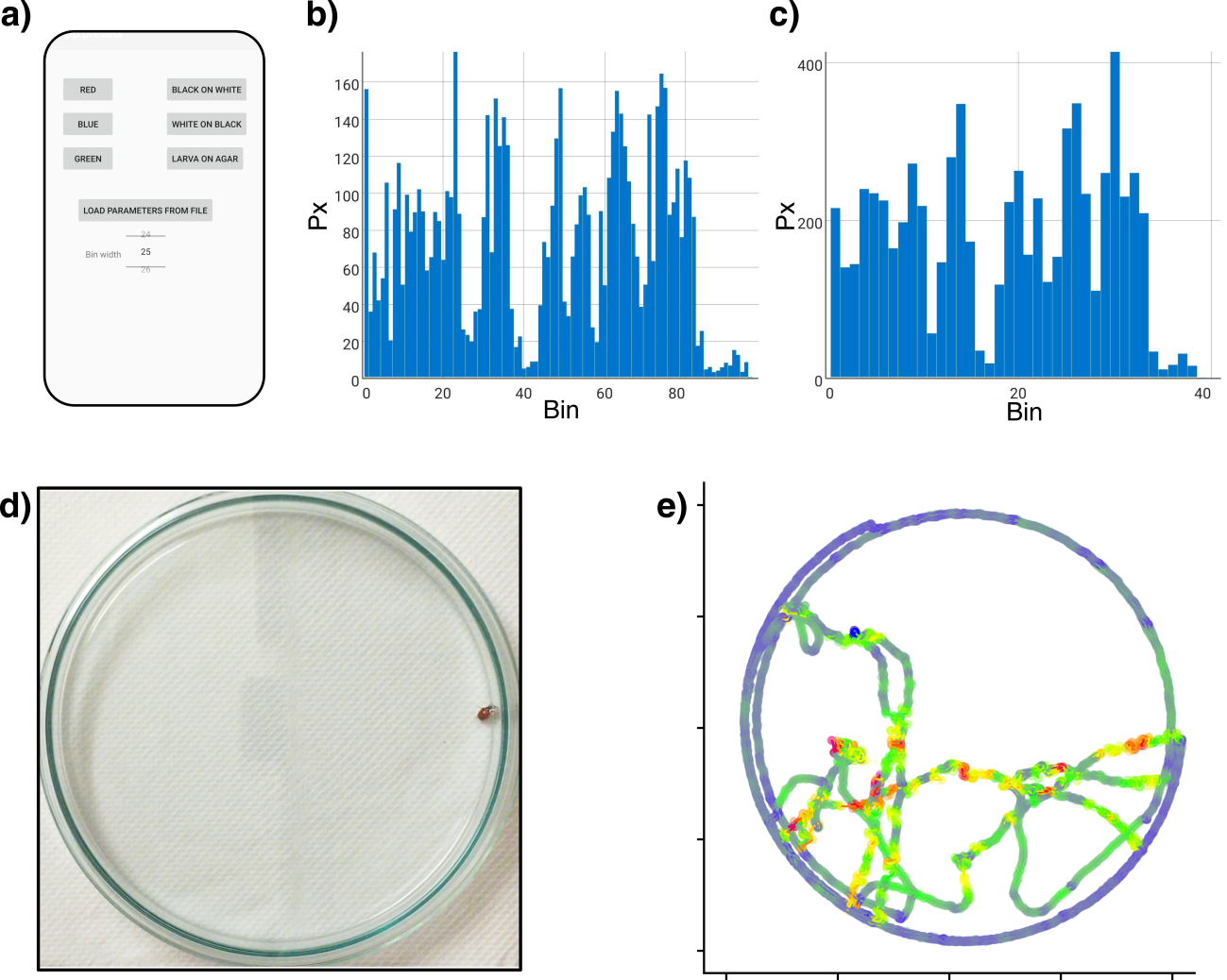


**Supplementary Figure 1.** (a) Presets menu in AnimApp (android version), showing preset threshold selections, option to load presets from file, and bin width setting for output (measured in frames). Locomotion of adult *Drosophila* (same source data as Figure 2e,f) with output in (b) 10 frame bins and (c) 25 frame bins, with distance measured in pixels (Px). These plots are generated within AnimApp and are directly shareable from within the app. (d) Preview frame of Harlequin Ladybird video and (e) tracked path, with relative instantaneous velocity indicated by colour.


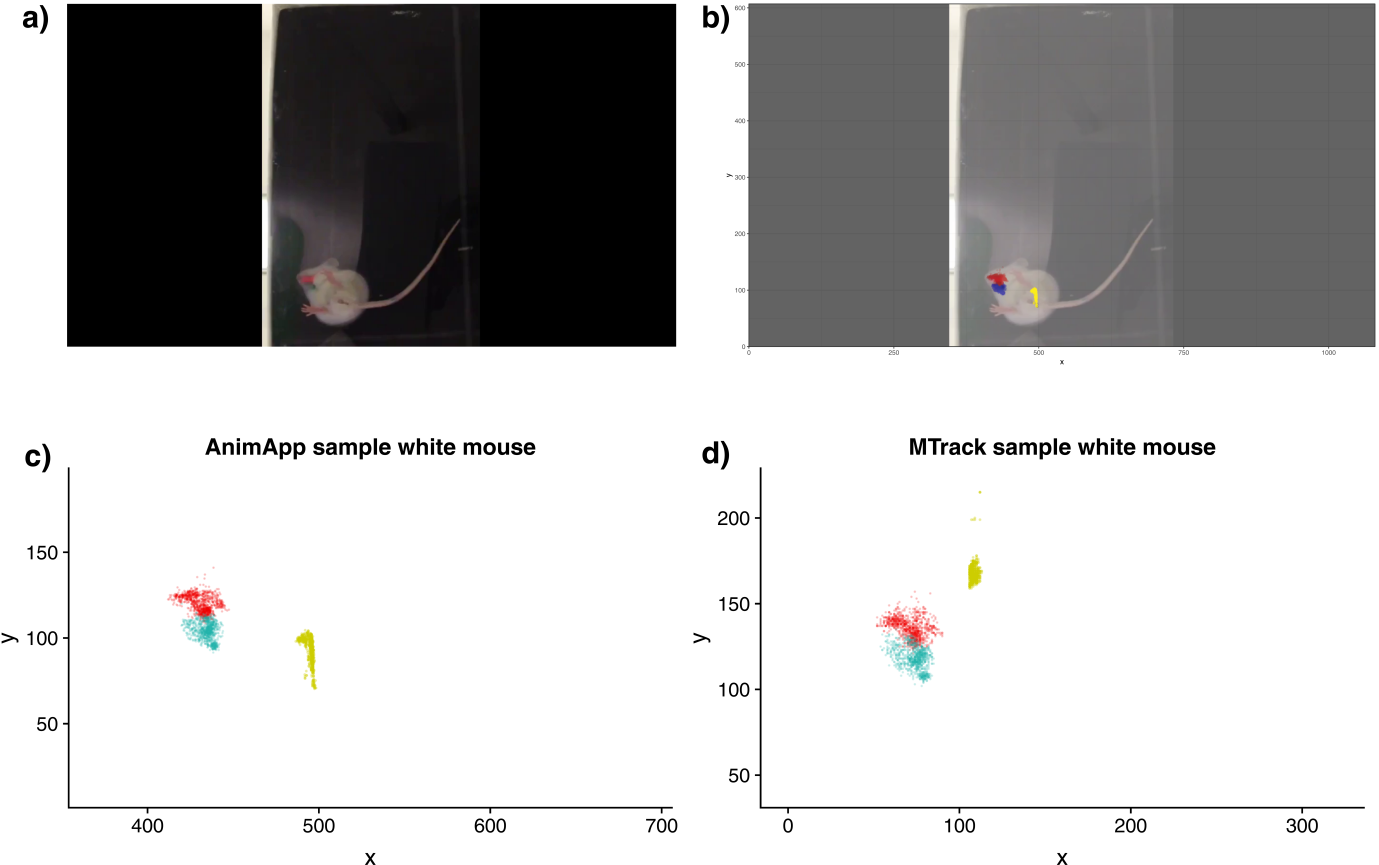


**Supplementary Figure 2.** (a) Preview frame of an example video from M-Track documentation ([*https://github.com/scimemia/M-Track*](https://github.com/scimemia/M-Track)), tracking a white mouse with coloured paws. (b) Overlay of AnimApp output for right paw (red), left paw (blue) and whole mouse body (yellow). Note that video frames have to be vertically flipped in order to overlay with plotted paths, since video frame axes start at the top left of image, while ggplot2 axes start at the bottom left of the graph. This reverses the apparent paw side between right and left. (c) Zoom of each paw path and location as detected by AnimApp and (d) example results provided for M-Track in GitHub documentation linked above. Left and right (blue and red points respectively) paw detection is highly similar between the two programs, with lower spread of points in the AnimApp data. Threshold setting for the mouse body (yellow points) in AnimApp appears to be more accurate than for M-Track, which may have also tracked the mouse’s reflection.

## Supplementary Files

*fly.mp4*

Example video of adult Drosophila moving in clear tube, as used for Figures 2e, 2f, Supplemental Figures 1d and 1e. Also available from [*https://github.com/sraorao/AnimApp/blob/master/fly.mp4*](https://github.com/sraorao/AnimApp/blob/master/fly.mp4)

*fly.mp4.settings.txt*

Example HSV settings file, for import to analyse fly.mp4, available from [*https://github.com/sraorao/AnimApp/blob/master/fly.mp4.settings.txt*](https://github.com/sraorao/AnimApp/blob/master/fly.mp4.settings.txt)

*fly_plot.R*

Example script for plotting path with trace colour derived from instantaneous velocity, available from [*https://github.com/sraorao/AnimApp/blob/master/fly_plot.R*](https://github.com/sraorao/AnimApp/blob/master/fly_plot.R)
